# Translational bioinformatics in the cloud: an affordable alternative

**DOI:** 10.1186/gm172

**Published:** 2010-08-06

**Authors:** Joel T Dudley, Yannick Pouliot, Rong Chen, Alexander A Morgan, Atul J Butte

**Affiliations:** 1 Program in Biomedical Informatics, Stanford University School of Medicine, 251 Campus Drive, Stanford, CA 94305, USA; 2 Department of Pediatrics, Stanford University School of Medicine, 300 Pasteur Drive, Stanford, CA 94305, USA; 3 Lucile Packard Children's Hospital, 725 Welch Road, Palo Alto, CA 94304, USA

## Abstract

With the continued exponential expansion of publicly available genomic data and access to low-cost, high-throughput molecular technologies for profiling patient populations, computational technologies and informatics are becoming vital considerations in genomic medicine. Although cloud computing technology is being heralded as a key enabling technology for the future of genomic research, available case studies are limited to applications in the domain of high-throughput sequence data analysis. The goal of this study was to evaluate the computational and economic characteristics of cloud computing in performing a large-scale data integration and analysis representative of research problems in genomic medicine. We find that the cloud-based analysis compares favorably in both performance and cost in comparison to a local computational cluster, suggesting that cloud computing technologies might be a viable resource for facilitating large-scale translational research in genomic medicine.

## Background

The intensely data-driven and integrative nature of research in genomic medicine in the post-genomic era presents significant challenges in formulating and testing important translational hypotheses. Advances in high-throughput experimental technologies continue to drive the exponential growth in publicly available genomic data, and the integration and interpretation of these immense volumes of data towards direct, measureable improvements in patient health and clinical outcomes is a grand challenge in genomic medicine. Consequently, genomic medicine has become rooted in and enabled by bioinformatics, engendering the notion of translational bioinformatics [1]. Translational bioinformatics is characterized by the challenge of integrating molecular and clinical data to enable novel translational hypotheses bi-directionally between the domains of biology and medicine [2,3]. In addition to the scientific challenges, the dimensionality and scale of genomic data sets presents statistical challenges, and also technical hurdles in gaining access to the computational power necessary to test even simple translational hypotheses using genomic data. For example, public data repositories such as the NCBI Gene Expression Omnibus (GEO) [4] enable researchers to ask novel and important translational questions such as, 'Which genes are most likely to be up-regulated specifically in cancers compared to all other human diseases' [5]? Given that GEO contains hundreds of thousands of clinical microarray samples, each with tens of thousands of gene abundance measurements, even a straightforward analysis of these data could require many billions or even trillions of comparisons.

While some of these challenges may be overcome by sophisticated computational techniques, raw computational power remains a substantial requirement that limits the conduct of such analyses. Although the cost of computing hardware has decreased substantially in recent years, investments of tens or hundreds of thousands of dollars are typically required to build and maintain a substantial scientific computing cluster. In addition to the hardware costs, sophisticated software to enable parallel computation is typically required, and staff must be hired to manage the cluster. Finally, substantial expenditures are required to pay for the utilities (for example, electricity, cooling) required for cluster operation. In this way, the computational requirements of contemporary genomic medicine are limiting, because access to the necessary computing power is restricted to those with the individual or institutional resources needed to install and maintain the necessary computational infrastructure. This unfortunately restricts the manner and scope of translational hypotheses that could otherwise be formulated and tested by researchers who do not have access to the necessary computational resources. Outside of clinical science, many organizations are exploring or using cloud computing technology to fulfill computational infrastructure needs.

Cloud computing potentially offers an efficient and economical means to obtain the power and scale of computation required to facilitate large-scale efforts in translational data integration and analysis. The definition of cloud computing itself is not concrete due to the many commercial interests involved. For the purposes of this article, we define cloud computing as 'a style of computing in which dynamically scalable and often virtualized resources are provided as a service over the Internet' [6]. Cloud computing is enabled by many technologies, but key among them is virtualization technology, which allows entire operating systems to run independently of the underlying hardware [7]. In most cloud computing systems, the user is given access to what appears to be a typical server computer. However, the server is really just a virtual 'instance' running at any one point on a large underlying hardware architecture, which is made up of many independent CPUs and storage devices. Viewed from an economic standpoint, cloud computing can be understood as a utility, much like water or electricity, where you only pay for what you use. In this sense, cloud computing provides access to a computational infrastructure on an on-demand, variable cost basis, rather than a fixed cost capital investment into physical assets.

Here, we present a case study evaluating the use of cloud computing technologies for a translational bioinformatics analysis of a large cancer genomics data set composed of matched replicate SNP genotype and gene expression microarray assay samples for 311 cancer cell lines, comprising 929 gene expression microarray samples and 622 SNP genotype array samples. We suggest that the data analysis illustrated by this case study is characteristic of computational challenges that might be faced by modern clinical researchers who have access to inexpensive high-throughput genomic assay technologies for profiling their patient populations. Our goal was to perform a statistical analysis to uncover expression quantitative trait loci (eQTL; that is, genomic loci associated with gene transcript abundance) that are common across cancer types. This entailed a statistical analysis whereby the genotype of each measured SNP was tested against the expression levels of each measured gene expression probe. The SNP platform used to generate our data measured 500,568 SNPs, and the gene expression microarray platform measured gene expression levels across 54,675 probes, requiring statistical evaluation of more than 13 × 10^9 ^comparisons. We estimated that it would take a single, modern server-class CPU more than 5,000 days to complete the analysis. Here we demonstrate the computational and economical characteristics of conducting this analysis using a cloud-based service, and contrast these characteristics with the computational and economic characteristics of performing the same analysis on a local institutional cluster.

## Methods

### Data

We downloaded the gene expression and genotyping data of 311 cancer cell lines from caBIG [8]. The mRNA expression of 54,675 probes in 929 samples was measured on the Affymetrix U133 Plus 2.0 platform. The genotypes of 500,568 SNPs in 622 DNA samples were measured on the Affymetrix 500K platform and analyzed using the oligo, pd.mapping250k.nsp, pd.mapping250k.sty R libraries in the Bioconductor [9].

### Cloud computing setup

Amazon Web Services (AWS) [10] elastic compute cloud (EC2) computing service was used for the analysis. EC2 instances were managed using the free edition of the RightScale Cloud Management Platform [11]. This tool was chosen because it provides visual interfaces for managing the cloud servers and executing scripts, which would be a plausible scenario for an investigator that lacked advanced computational abilities. All virtual instances used in the analysis were of the m1.large EC2 instance type [12] running 64-bit CentOS Linux version 5.2 [13]. This instance type was chosen because it was determined to be the most economical choice given the amount of system memory required (>12 GB) by the analysis. A total of 100 EC2 instances were used for the analysis. One of these instances served as the job control and data-partitioning server. This server used the MySQL relational database server v.5.1 [14] to store accounting and job control data pertaining to the execution start and stop times of each compute node, as well as the comparison indices issued to each compute node. The compute nodes were provisioned using the RightScale dashboard using a custom startup script that installed the required version of the R statistical computing environment, as well as additional R packages upon server initialization. In particular, the RMySQL package [15] was used to communicate with the database running on the data-partitioning server, and the 'ff' package [16] was used to store data partitions as memory-mapped, disk-based data frames to enable efficient use of compute node system memory.

### Local cluster setup

We used a dedicated 240 core High Performance Compute Cluster based on the Hewlett Packard C-class BladeSystem attached to 15 TB storage area network. Each compute node has dual socket quad-core Intel E5440 Harpertown CPUs for a total of 8 processors per node with 16 GB of ram and interconnected with 4 × DDR InfiniBand switched fabric. The cluster uses the Platform HPC Workgroup Manager cluster operating system with Platform LSF cluster distributed workload management. The cluster is hosted in a water-cooled rack at Stanford ITS Forsythe data center, a secure monitored facility with uninterruptible power supply (UPS) and standby backup power generators. The analysis was restricted to 198 of the 240 available CPUs to enable an equitable comparison with the cloud-based analysis.

### Statistical analysis

All statistical analysis were performed using the R statistical computing environment [17]. Putative eQTLs were evaluated using a one-way analysis of variance (ANOVA) test. For each SNP-expression probe pair, we grouped the expression values for that probe across all samples according to their respective genotype for the SNP as denoted by homozygous major (AA), homozygous minor (aa) and heterozygous (Aa). Using the genotype designations as factors, we carried out a one-way ANOVA to test the null hypothesis that the means of the expression levels across all three genotype categories were equal. *P*-values from the one-way ANOVA were corrected using the Bonferroni method. If the one-way ANOVA rejected the null hypothesis after correction, we determined that the SNP was an eQTL for the particular expression probe.

### Cost estimation

Costs for the local cluster were estimated by spreading capital costs of hardware and software over a 3-year period, representing the typical service lifetime of computer hardware in academic research. Per-year operational costs were projected assuming a 5% cost inflation rate each year. An average yearly cost was estimated from the total capital and operational costs estimated over the 3-year period, and from this we computed an hourly cost for operating the cluster, which was divided by the number of CPUs in the cluster to estimate the per-CPU/per-hour cost of operating the local cluster.

## Results

**Figure 1:**
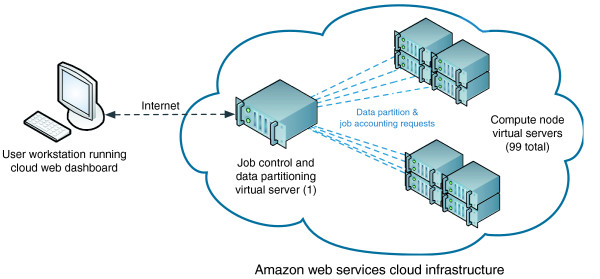
**Schematic illustration of the computational strategy utilized for the cloud-based eQTL analysis**. One hundred virtual server instances are provisioned using a web-based cloud control dashboard. One of the virtual server instances served as a data distribution and job control server. Upon initialization, the compute nodes would request a subset partition of eQTL comparisons and insert timestamp entries into a job accounting database upon initiation and completion of the eQTL analysis subset it was administered.

From our data set of matched pairs of 622 SNP genotype arrays and 929 gene expression microarrays assayed as matched pairs across 311 cancer cell lines, we evaluated 13,029,271,200 SNP-expression probe pairs to evaluate if any of the SNPs could be considered as eQTLs based on experimental measurements across all samples. Each pair-wise comparison comprised approximately 700 genotype versus expression data points, thereby generating >9.0 × 10^12 ^total data points. The total set of pair-wise SNP-expression comparisons was broken into 99 equal subsets, which were evaluated in parallel across 99 individual compute node instances. One additional server instance served as the data and index server that distributed the comparison sets to each node, and also collected operational statistics (for example, eQTL analysis start/stop times) from each of the compute nodes (Figure 1). Each compute node executed two separate eQTL analysis processes that ran in parallel. Each process performed eQTL analysis on one of the data subsets, evaluating 131 × 10^6 ^SNP-expression probe pairs in sequence. Under this scheme, the analysis was distributed across 198 computational processes executing across 99 compute node instances in the cloud infrastructure. This computational strategy was executed on the AWS [10] EC2 infrastructure using virtual server instances, and also on our local institutional compute cluster with similar operating system specifications to the EC2 instances. The analysis was restricted to use only 198 of the 240 available CPU cores on the local cluster to allow for an equitable performance comparison.

The eQTL analysis completed in approximately 6 days on both systems (Table 1), with the local cluster completing the computation 12 hours faster than the virtual cloud-based cluster. The total cost for running the analysis on the cloud infrastructure was approximately three times the cost of the local cluster (Table 2). The final results of the eQTL analysis yielded approximately 13 × 10^9 ^one-way ANOVA *P*-values, respective to the total number of SNP-expression probe pairs that were evaluated. After correcting the one-way ANOVA *P*-values using the Bonferroni method, 22,179,402 putative eQTLs were identified.

**Table 1:** Performance and economic metrics for eQTL analysis for cloud-based and local compute clusters

	eQTL analysis on AWS cloud	eQTL analysis on local cluster
Running time	6 days 0.1 hours	5 days 11.9 hours
Total CPUs	198	198
Cost per CPU	$0.19	$0.06
Total analysis cost	$5,417.28	$1,710.00

Per CPU costs for the local cluster were estimated using the cost structure detailed in Table 2.

**Table 2:** Cost structure used to estimate cost rate for local compute cluster CPUs

Category	Cost year 1	Cost year 2	Cost year 3	Total cost over 3 years	Average cost per year	Average cost per hour
Hardware and support	$56,667	$56,667	$56,667	$170,000	$56,667	
Software licensing	$5,000	$5,000	$5,000	$15,000	$5,000	
Server hosting	$23,424	$25,766	$28,343	$77,533	$25,844	
Personnel	$43,500	$45,675	$47,959	$137,134	$45,711	
**Entire cluster**						**$15.21**
**Per CPU @240 CPUs**						**$0.06**

Estimates are based on real-world costs associated with the local compute cluster used as the basis for comparison in this study. A per-CPU/per-hour cost was used as the basis for comparison with the cloud-based system.

## Discussion

Using a real-world translational bioinformatics analysis as a case study, we demonstrate that cloud computing is a viable and economical technology that enables large-scale data integration and analysis for studies in genomic medicine. Our computational challenge was motivated by a need to discover cancer-associated eQTLs through integration of two high-dimensional genomic data types (gene expression and genotype), requiring more than 13 billion distinct statistical computations.

It is notable that execution of our analysis completed in approximately the same running time on both systems, as it could be expected that the cloud-based analysis would take longer to execute due to possible overhead incurred by the virtualization layer. However, in this analysis, we find no significant difference in execution performance between a cloud-based or local cluster. This may be attributable to our design of the analysis code, which made heavy use of CPU and system memory in an effort to minimize disk input/output. It is possible that an analysis that required many random seeks on the disk could have realized a performance disparity between the two systems.

Although the total cost for running the analysis on the cloud-based system was approximately three times more expensive compared to the local cluster, we assert that the magnitude of this cost is well within reach of the research (operational) budgets of a majority of clinical researchers. There are intrinsic differences between these approaches that prevent us from providing a completely accurate accounting of costs. Specifically, we chose to base our comparison on the cost per CPU hour because it provided the most equivalent metric for comparing running-time costs. However, because we are comparing capital costs (local cluster) to variable costs (cloud), this metric does not completely reflect the true cost of cloud computing for two reasons: we could not use a 3-year amortized cost estimate for the cloud-based system, as done for the local cluster; and the substantial delay required to purchase and install a local cluster was not taken into account. As these factors are more likely to favor the cloud-based solution, it is possible that a more sophisticated cost analysis would bring the costs of the two approaches closer to parity.

There are several notable differences in the capabilities of each system that give grounds for the higher cost of the cloud-based analysis. First, there are virtually no startup costs associated with the cloud-based analysis, whereas substantial costs are associated with building a local cluster, such as hardware, staff, and physical housing. Such costs range in the tens to hundreds of thousands of dollars, likely making the purchase of a local cluster prohibitively expensive to many. It can take months to build, install and configure a large local cluster, and therefore there is also the need to consider the non-monetary opportunity costs incurred during initiation of a local cluster. The carrying costs of the local cluster that persist upon conclusion of the analysis should also be considered. The cloud-based system offers many technical features and capabilities that are not matched by the local cluster. Chief among these is the 'elastic' nature of the cloud-based system, which allows it to scale the number of server instances based on need. If there was a need to complete this large analysis in the time-span of a day, or even several hours, the cloud-based system could have been scaled to several hundred server instances to accelerate the analysis, whereas the local cluster size is firmly bound by the number of CPUs installed. A related feature of the cloud is the user's ability to change the computing hardware at will, such as selecting fewer, more powerful computers instead of a larger cluster if the computing task lends itself to this approach.

Other features unique to the cloud include 'snapshotting', which allows whole systems to be archived to persistent storage for subsequent reuse, and 'elastic' disk storage that can be dynamically scaled based on real-time storage needs. A feature of note that is proprietary to the particular cloud provider used here is the notion of 'spot instances', where a reduced per-hour price is set for an instance, and the instance is launched during periods of reduced cloud activity. Although this feature may have increased the total execution time of our analysis, it might also reduce the cost of the cloud-based analysis by half depending on market conditions. Clearly, any consideration for the disparities in the costs between the two systems must consider additional features and technical capabilities of the cloud-based system.

While we find that the cost and performance characteristics of the cloud-based analysis are accommodating to translational research, it is important to acknowledge that substantial computational skills are still required in order to take full advantage of cloud computing. In our study, we purposefully chose a less sophisticated approach of decomposing the computational problem by simple fragmentation of the comparison set. This was done to simulate a low-barrier of entry approach to using cloud computing that would be most accessible to researchers lacking advanced informatics skills or resources. Alternatively, our analysis would likely have been accelerated significantly through utilization of cloud-enabled technologies such as MapReduce frameworks and distributed databases [18]. It should also be noted that while this manuscript was under review, Amazon announced the introduction of Cluster Computer Instances intended for high performance computing applications [19]. Such computing instances could further increase accessibility to high-performance computing in the cloud for non-specialist researchers.

There are serious considerations that are unique to cloud computing. Local clusters typically benefit from dedicated operators who are responsible for maintaining computer security. By contrast, cloud computing allows free configuration of virtual machine instances, thereby sharing the burden of security with the user. Second, cloud computing requires the transfer of data, which introduces delays and can lead to substantial additional costs given the size of many data sets used in translational bioinformatics. Users will need to consider this aspect carefully before adopting cloud computing. An additional data-related limitation we faced repeatedly with our provider was a 1-terabyte limit on the size of the virtual disks.

However, the most significant impediment facing biomedical researchers wishing to adopt cloud computing involves the software environment for designing the computing environment and running the experiments. We believe efforts for fully exposing the capabilities of cloud-computing environments at the application level are key to enhancing the democratizing effect of cloud computing in genomic medicine. Specifically, intuitive and scalable software tools are needed to enable clinician scientists at the forefront of medical discovery to leverage fully the vast resources of public data and cloud-based computing infrastructure. Cloud-based tools should be specifically oriented to address the particular modes of inquiry of clinician scientists towards enabling unified biological and clinical hypothesis evaluation. Rather than present the clinical investigator with a collection of bioinformatics tools (that is, the 'toolbox' approach), we believe clinician-oriented, cloud-based translational bioinformatics systems are key to facilitating data-driven translational research using cloud computing.

It is our hope that by demonstrating the utility and promise of cloud computing for enabling and facilitating translational research, investigators and funding agencies will commit efforts and resources towards the creation of open-source software tools that leverage the unique characteristics of cloud computing to allow for uploading, storage, integration and querying across large repositories of public and private molecular and clinical data. In this way, we might realize the formation of a biomedical computing commons, enabled by translational bioinformatics and cloud computing, that empowers clinician scientists to make full use of the available molecular data for formulating and evaluating important translational hypotheses bearing on the diagnosis, prognosis, and treatment of human disease.

## Abbreviations

ANOVA: analysis of variance; AWS: Amazon Web Services; CPU: central processing unit; EC2: elastic compute cloud; eQTL: expression quantitative trait loci; GEO: Gene Expression Omnibus; SNP: single nucleotide polymorphism.

## Competing interests

The authors declare that they have no competing interests.

## Authors' contributions

AJB conceived of the study. AJB, JTD and YP designed the study. JTD and YP designed and carried out the analysis. RC and AJM prepared the eQTL data for analysis. AJB, JTD and YP wrote the paper.
